# Understanding limited effectiveness of a set of interventions implemented under continuous quality improvement methodology: a process assessment informed by MRC guidance

**DOI:** 10.3389/frhs.2026.1884875

**Published:** 2026-07-15

**Authors:** Kelly Estrada-Orozco, Steffany Villate-Soto, Giancarlo Buitrago, Marcela Bejarano-Villamarin, Cristian Camilo Veloza-García, Valeria Herrera-Villamizar, Oscar Dueñas, Jairo Morantes-Caballero, Javier Eslava-Schmalbach, Rodrigo Pardo, Hernando Gaitán-Duarte

**Affiliations:** 1 Fundación Universitaria Sanitas, Bogotá, Colombia; 2 Evidence and Implementation Center (CEI-Evidence). Bogotá, Colombia; 3 Clinical Research Institute, School of Medicine, Universidad Nacional de Colombia, Bogotá, Colombia; 4 Hospital Universitario Nacional de Colombia, Bogotá, Colombia

**Keywords:** continuous quality improvement, medication safety, patient safety, process assessment, surgery

## Abstract

**Introduction:**

Medication errors remain a major contributor to adverse events (AEs) in surgical services, particularly in low-middle income countries (LMICs), where structural and organizational constraints may limit the effectiveness of quality improvement strategies. Continuous quality improvement (CQI) methods are widely implemented to reduce preventable harm; however, evidence regarding their real-world effectiveness and the mechanisms underlying success or failure in LMIC contexts is limited. This study aimed not only to evaluate the impact of a set of interventions under CQI methodology targeting medication prescribing and administration processes, but also to explain the absence of expected effects through a process-informed assessment.

**Methods:**

A prospective cohort study using an interrupted time series design without a control group was conducted in a high-complexity teaching hospital in Bogotá, Colombia. The intervention targeted prescribing and administration of antibiotics, anticoagulants, and opioids, and included protocol reinforcement, a mobile application (PROA UNAL), standardized pain assessment, and three Plan-Do-Study-Act cycles. Process and outcome indicators were measured biweekly across baseline, implementation, and sustainability phases. Segmented regression and multivariable logistic regression assessed intervention effects, while a structured process assessment examined fidelity, mechanisms of impact, and contextual factors.

**Results:**

A total of 713 patients were distributed across the three phases of the study (194 baseline; 366 implementation; 153 sustainability). The three structured PDSA cycles were conducted, starting from protocol development to the adherence monitoring for each intervention. Implementation fidelity varied across components (100% for prophylactic antibiotic timing, 80% for PROA UNAL application use, and 40% for standardized postoperative pain scale implementation). No significant improvements were observed in process indicators, medication-related AEs, or clinical incidents (CIs). Adjusted models confirmed the absence of intervention effects. Process evaluation identified key contextual barriers—high staff turnover, fragmented governance, limited frontline engagement, and lack of real-time feedback—that hindered implementation.

**Discussion:**

The absence of measurable effects appears attributable to limited fidelity and contextual misalignment rather than intrinsic ineffectiveness of the interventions under the CQI methodology. Future initiatives in LMICs surgical settings should incorporate readiness assessments, governance alignment, real-time feedback, and sustained frontline.

## 1 Introduction

Medication errors are defined as “any preventable event that may cause or lead to inappropriate medication use or patient harm while the medication is in the control of the healthcare professional, patient, or consumer” (1). They are particularly frequent in surgical services (2, 3), where care processes are complex, involve multiple professionals, and frequently include high-alert medications (4). Adverse events (AEs) related to medication errors represent a substantial proportion of preventable harm in hospitalized surgical patients (5).

Medication use in hospitals involves multiple sequential steps: prescribing, verification, dispensing, administration, monitoring, and reporting (1). Errors frequently originate in prescribing and may propagate into administration errors, potentially leading to adverse drug events (ADEs) (6). Although evidence suggests that medication administration errors and potential ADEs are common (7), literature describing the effectiveness of interventions under continuous quality improvement (CQI) initiatives in reducing such outcomes in surgical services, particularly in low-middle income countries (LMICs) contexts, remains scarce.

Clinical risk management systems (CRMS) aim to detect, monitor, evaluate, and prevent patient harm (8). Among these, CQI methods represent structured, iterative approaches to improving healthcare processes (9). However, evidence suggests that the success of interventions derived from CQI depends not only on methodological rigor but also on adequate implementation fidelity and contextual alignment (10, 11). LMICs health systems face additional structural challenges, including workforce instability, limited technological infrastructure, and resource constraints; that may influence intervention effectiveness (12).

At the Hospital Universitario Nacional de Colombia, 11.4% of surgical patients experienced AEs in 2017, with 33% associated with medication errors (13). Building upon previous successful CQI experiences in non-surgical services (14), a multidisciplinary team implemented a set of interventions under the CQI methodology, targeting prescribing and administration processes in surgical services. The implementation of this method included (1) root cause analysis to identify causes of problems, (2) iterative testing of intervention(s), (3) evaluation of the process and (4) outcome measures over time.

Complex interventions may fail to demonstrate effectiveness not because their theoretical foundation is flawed, but because implementation fidelity is insufficient or contextual factors prevent activation of intended mechanisms of change. Understanding such implementation failures is essential to advance health services research and improve patient safety strategies in LMICs settings.

The objective of this study was to evaluate the implementation and effectiveness of a set of interventions under the CQI methodology aimed at improving prescribing and administration processes in surgical services, and to explain the absence of expected clinical effects through a structured process assessment.

## 2 Materials and methods

### 2.1 Study design

We conducted a prospective cohort study using an interrupted time series (ITS) design without a control group. The ITS approach allows evaluation of level and trend changes while accounting for underlying temporal patterns (11).

### 2.2 Setting and participants

The study was conducted in a high-complexity adult teaching hospital in Bogotá, Colombia, with 80 surgical beds and approximately 620 surgeries per month. The hospital provides care to patients covered by contributory and subsidized insurance regimes under the Colombian health system.

Healthcare professionals involved included surgical specialists, residents, anesthesiologists, and nursing staff working in operating rooms, recovery areas, and surgical wards.

Patients aged ≥18 years hospitalized at least 12 h in surgical services and receiving at least one dose of antibiotics, anticoagulants, or opioids were eligible. Exclusion criteria included prior AEs in other institutions, minor procedures, and pregnancy.

### 2.3 Intervention theory and components

Based on root cause analysis, contributing factors included poor adherence to protocols, inadequate communication, and information system limitations (Supplementary Figure S1). The intervention aimed to reduce medication-related AEs and medication-related clinical incidents (CIs), by activating three mechanisms:

1. Improving adherence to evidence-based prescribing protocols.
2. Enhancing decision support through mobile access to institutional guidelines (PROA UNAL).
3. Standardizing postoperative pain assessment to guide opioid use.

**Figure 1:**
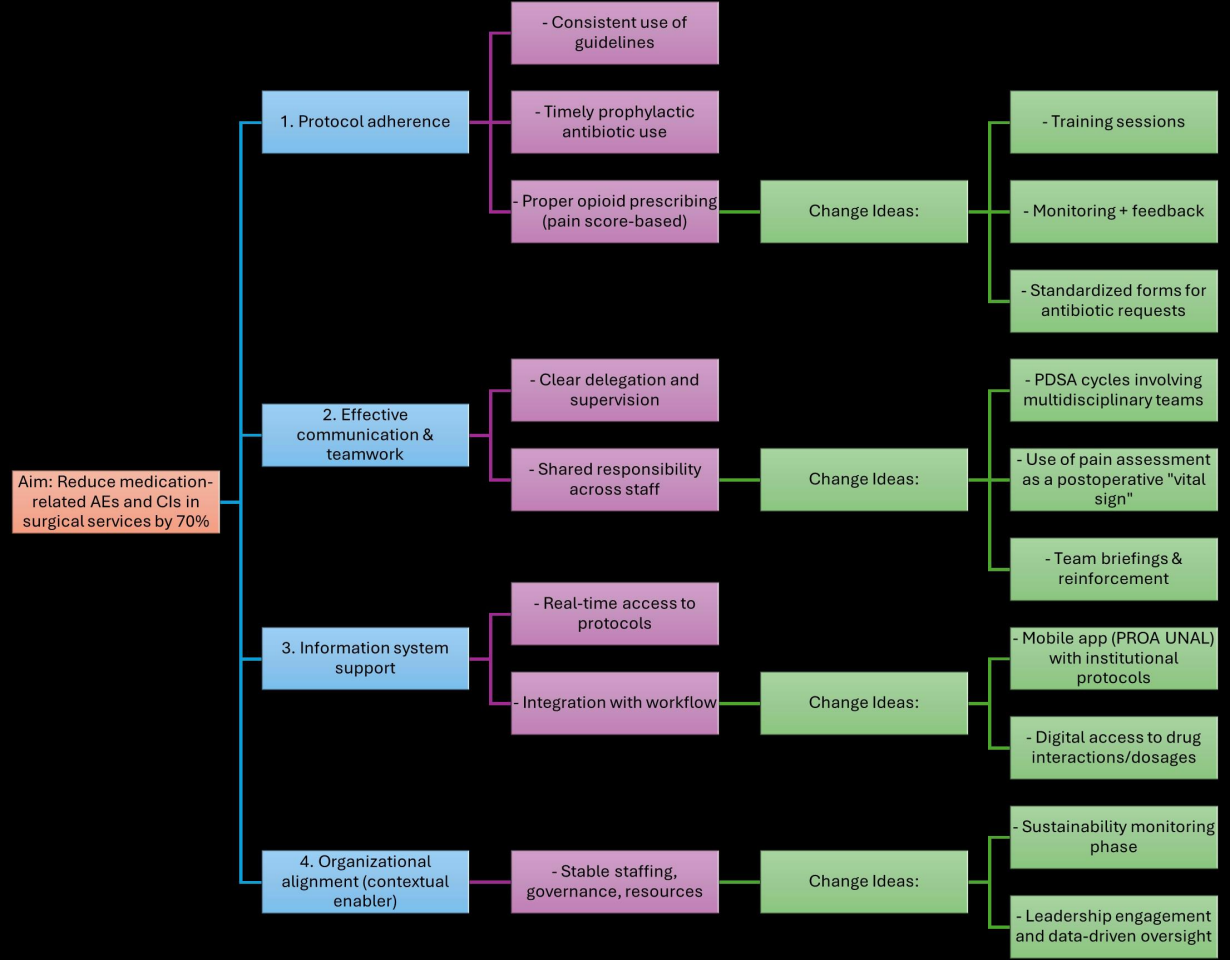
Driver diagram of the implemented interventions.

The set of interventions included (Figure 1):

- Reinforcement of prophylactic antibiotic timing (30–60 min before incision).
- Implementation of the PROA UNAL mobile application.
- Standardized postoperative pain assessment using validated scales.
- Mechanisms to implement the interventions under CQI methodology:
- Multidisciplinary improvement team was conformed
- Structured training for the improvement team

Development of three Plan-Do-Study-Act (PDSA) cycles.This study consisted of three phases: the baseline phase lasted five biweekly periods (July–September 2022), followed by eleven implementation biweekly periods (October 2022–March 2023), and a sustainability phase (October–December 2023). During all phases, an investigator was assigned to review the daily inpatient census for surgical services. From this census, a simple random sample of six patients was selected daily, and the first four who met the inclusion criteria were enrolled. Patient records were reviewed at discharge—whether due to improvement, transfer, or death—to identify potential prescribing or administration errors, as well as any AEs and CIs, using data from medical prescriptions, anaesthesia records, nursing notes and medication administration charts.

### 2.4 Process assessment domains

To interpret the effectiveness findings and explain the absence of expected impact, we conducted a process assessment informed by the Medical Research Council (MRC) guidance for evaluating complex interventions (15, 16). The MRC framework conceptualizes process assessment as a structured evaluation of how an intervention is implemented (implementation fidelity, dose and adaptations), how it produces change (mechanisms of impact), and how contextual factors influence both implementation and outcomes. This framework is particularly suited for complex, multicomponent quality improvement interventions delivered in dynamic healthcare settings, where outcomes may be shaped not only by intervention design but also by organizational structures, workforce stability, and system readiness. We selected the MRC guidance because it provides a theoretically grounded and widely adopted structure to examine the interaction between implementation processes and contextual constraints, thereby allowing a rigorous interpretation of null or modest effectiveness findings. In LMICs hospital settings, where structural and governance challenges may substantially influence intervention delivery, such an explanatory approach is critical to distinguish between intervention failure and implementation failure (16).

All the data for the process assessment were obtained from two sources: (1) informal interviews to the implementator team and (2) results from the barriers and facilitators related to the implementation of interventions, using surveys to the staff (17). Those data were collected for an independent researcher who did not participate in the implementation of the interventions derived from CQI project.

To explain intervention effects, we assessed:

- **Implementation fidelity**: extent to which each component was delivered as intended.
- **Dose delivered and uptake**: proportion of intended exposure achieved.
- **Mechanisms of impact**: whether process indicators changed as expected.
- **Contextual influences**: organizational and structural factors affecting implementation.

### 2.5 Variables measured

Sociodemographic characteristics of the patients included in the study were collected, including age group, and type of affiliation to the general Colombian social security system in Colombia (state-subsidised, workers' contributory system). Clinical characteristics included weight (kg), height (cm), main diagnosis upon admission, Charlson index score, length of hospital stay, treatment unit and number of medications administered at the time of study inclusion.

### 2.6 Outcomes

For this study, the primary outcome was a description of the implementation process. Implementation fidelity was assessed through a survey administered to staff members, which evaluated the degree to which the intervention was delivered as originally designed and whether any adaptations were made during implementation. Further details on the survey findings have been published previously by the study group (17).

Other outcomes measured from the CQI included process indicators and primary clinical outcomes.

Process indicators included correct prescribing and administration of prophylactic antibiotics and thromboprophylaxis, and pain scale documentation (Supplementary Table S1).

Primary clinical outcomes were medication-related AEs and medication-related CIs, defined according to established criteria (18, 19) and previously validated methodology (3).

### 2.7 Statistical analysis

Descriptive statistics were calculated for clinical and sociodemographic variables. Comparisons across phases used Chi-square and Mann–Whitney tests.

Biweekly AEs and CIs proportions were analyzed using segmented regression with Newey–West standard errors, regarding the baseline and implementation phases. Model assumptions were verified using correlograms, Bartlett test, periodograms, Cumby–Huizinga test, and Durbin–Watson adjustments.

Multivariable logistic regression models estimated odds ratios (OR) and adjusted odds ratios (aOR) for AEs and CIs, controlling for clinical and sociodemographic variables.

Analyses were performed using R® and Stata®.

## 3 Results

### 3.1 Implementation fidelity and dose delivered

**Figure 2:**
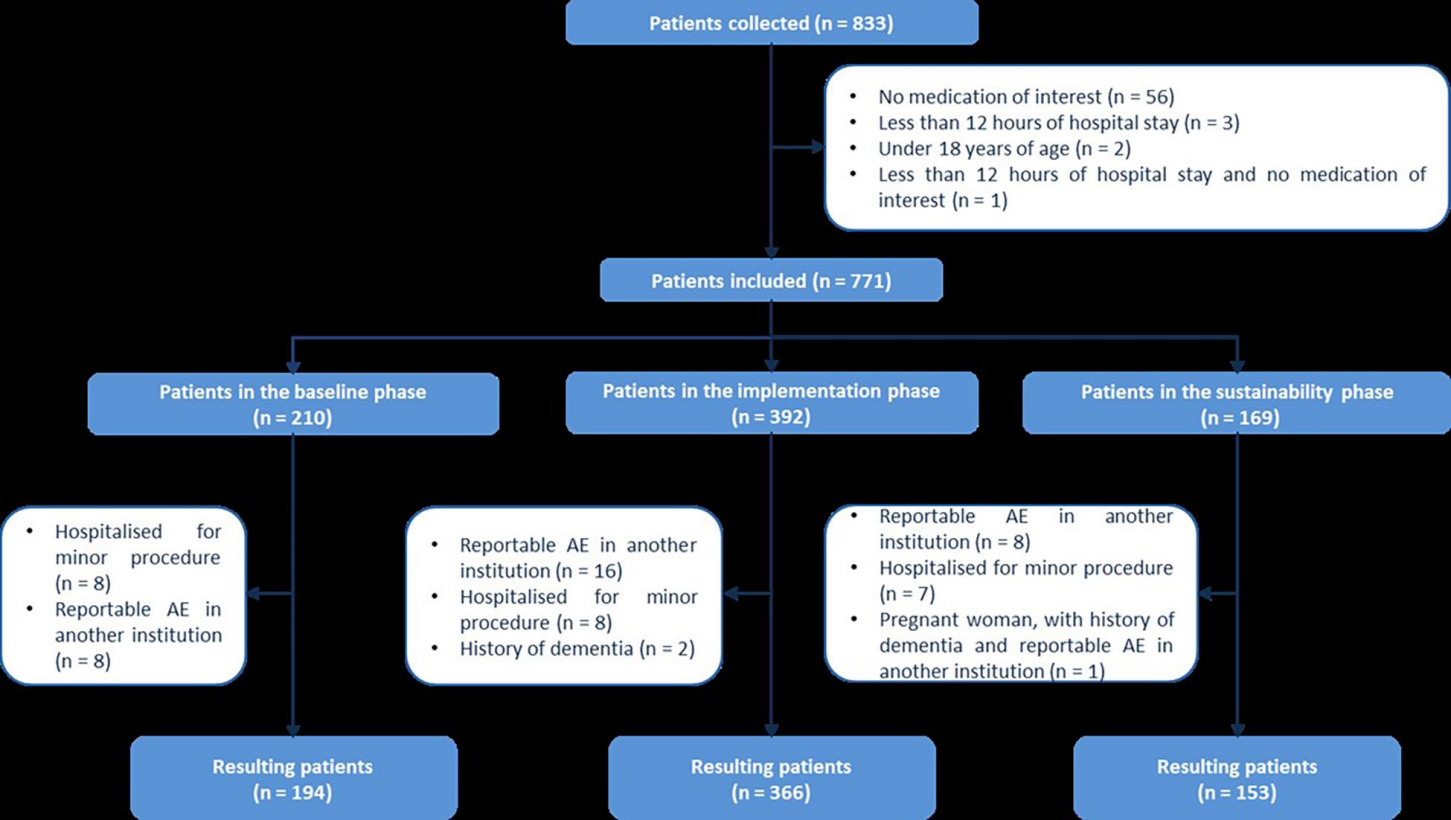
Flowchart of the patient inclusion and exclusion process.

A total of 833 patients were randomly sampled, of whom 713 met inclusion criteria and completed follow-up across study phases (194 baseline; 366 implementation; 153 sustainability) (Figure 2).

Based on the three PDSA cycles (Table 1), the set of interventions under the CQI methodology followed a progressive implementation logic: each cycle moved from protocol development and initial training toward systematic adherence monitoring and institutional consolidation, reflecting an iterative refinement process.

**Table 1:** Application of PDSA cycles to interventions derived from the use of the CQI method.

Cycle\Interventions		Reinforcement of prophylactic antibiotic timing (30–60 min before incision)	PROA UNAL mobile application	Standardized postoperative pain assessment using validated scales
Cycle 1	Plan	To ensure timely antibiotic prescription prior to patient arrival at the operating theater.	To update institutional guidelines on the prescription and administration of prioritized medications in surgical settings and to provide rapid, easy access to them through a mobile application.	To standardize the use of a validated pain scale for postoperative opioid prescribing and to define score thresholds for initiating opioid therapy.
	Do	Adherence to the antibiotic prescription protocol was monitored and implementation was verified.	Guideline revision teams were set up.	The pain assessment tool was developed and healthcare staff were trained in its use.
			Management flow diagrams were created and incorporated into the mobile application.	
	Study	Adherence to the intervention reached 90%, indicating successful initial uptake of the protocol.	Adherence to the application and its usability among prescribers were assessed. This adherence was low given the complexity of the mobile application interface.	The pain assessment tool was designed to provide a continuous score, which made it difficult for surgery staff to reach a consensus. Therefore, the following categories were created to simplify the measurement: Mild (scores 1–3) Moderate (scores 4–7) Severe (scores 8–10)
	Act	Ongoing monitoring of adherence to the antibiotic timing protocol was established and incorporated into routine surgical checklist procedures.	A simplified interface was designed to streamline access to guideline recommendations within the mobile application.	A pilot test was conducted.
Cycle 2	Plan	To evaluate sustained adherence to the prophylactic antibiotic timing protocol and to identify persistent gaps in compliance.	To implement and verify the use of the mobile application.	To evaluate the adherence of the use of the pain assessment tool.
	Do	Prescription document review was carried out.	Use of the mobile application by prescribers was verified.	Adherence to the use of the tool was assessed and its use was reinforced among the medical staff. Discussions with the surgery staff to formalize its use.
	Study	Adherence to the protocol and its implementation were assessed, confirming consolidation of the timing standard.	Adherence to the use of the mobile application among prescribers was assessed. Adherence increased.	Usability and adherence of the pain assessment tool use were evaluated.
				The surgery staff mostly used the tool. However, the need to simplify the measurement of process indicators was identified, for which two additional categories were consolidated: Score less than 8 Score greater than or equal to 8
	Act	The antibiotic timing protocol was formally incorporated into institutional surgical safety processes and standardized across surgical units.	A pilot test of the mobile application prototype was carried out and adjustments made.	The importance of systematic postoperative pain assessment was reinforced with nursing staff in recovery wards to improve consistent use of the scale.
Cycle 3	Plan	—	To ensure ongoing training of prescribing physicians and residents and to establish continuous updating of application content in line with current guidelines.	To train newly hired staff in the use of the pain assessment tool and to reinforce its clinical value among nursing personnel in recovery wards.
	Do		Dissemination sessions and training on the mobile application were carried out with hospital physicians and medical residents.	Newly hired staff in recovery wards were trained in the use of the pain assessment tool, and continuous follow-up of its application was maintained.
	Study		Usability of the application was assessed through user surveys administered to prescribers.	Process indicators for the pain assessment tool were assessed to evaluate the degree of uptake across the surgical service.
	Act		The mobile application was formally incorporated into the hospital's prescribing procedures as a standard clinical decision-support tool.	Uninterrupted training, emphasizing the importance of using the tool.

Although structured PDSA cycles were conducted and feedback was provided to clinical teams, full integration of all intervention components into routine workflows was not achieved.

Implementation fidelity varied substantially across intervention components:

- Prophylactic antibiotic timing reached 100% implementation.
- The PROA UNAL mobile application achieved approximately 80% implementation, limited by uptake and adherence issues.
- Standardized postoperative pain scale documentation reached only 40%.

These findings indicate partial delivery of the intervention package and heterogeneous uptake across components.

### 3.2 Activation of mechanisms of impact

The intervention theory assumed that improved adherence to prescribing protocols, enhanced decision support, and standardized pain assessment would modify prescribing and administration behaviors, thereby reducing medication-related AEs and CIs.

However, analysis of process indicators showed:

- No sustained shifts in median compliance proportions (Table 2).
- No special cause variation in control charts (Supplementary Figures S2–S7).
- No significant trend changes across baseline, implementation, and sustainability phases.

**Table 2:** Median compliance proportion of the process indicators, for each phase.

Process indicators	Baseline *n* = 194 *n* (%)	Implementation *n* = 366 *n* (%)	*p*-value	Sustainability *n* = 153 *n* (%)	*p*-value
Proportion of patients with correct prescription of prophylactic antibiotics					
Median (IQR)	100 (100.00–100.00)	93.33 (88.89–100.00)	0.13	100.00 (83.33–100.00)	0.86
Proportion of patients who received opportunely the prophylactic antibiotic					
Median (IQR)	95.00 (87.50–96.55)	94.44 (90.83–100.00)	0.69	100.00 (91.67–100.00)	0.68
Proportion of patients with correct prescription of thromboprophylaxis					
Median (IQR)	85.71 (85.71–88.24)	92.86 (86.06–97.62)	0.23	100.00 (92.86–100.00)	0.24
Proportion of patients who received thromboprophylaxis					
Median (IQR)	93.75 (93.75–94.44)	100 (92.33–100.00)	0.55	95.24 (93.75–95.83)	0.44
Proportion of patients with a VAS score of less than 4					
Median (IQR)	33.33 (30.43–48.00)	43.75 (42.50–50.00)	0.21	44.83 (23.81–45.45)	0.57
Proportion of patients with a VAS score greater than or equal to 4					
Median (IQR)	52.17 (36.67–54.17)	38.46 (34.51–44.65)	0.28	37.93 (32.35–50.00)	0.86

Thus, the intended proximal mechanisms of change (behavioral modification in prescribing and administration) were not consistently activated.

### 3.3 Patients' characteristics

The patients included in the study displayed similar characteristics across the baseline, implementation and sustainability phases in terms of age, sex, insurance affiliation, weight, height and Charlson comorbidity score. Differences were noted in length of hospital stay, treating unit and administration of two or more medications, which were more frequent during the implementation and sustainability phases (Table 3).

**Table 3:** Sociodemographic and clinical characteristics of the patients.

Sociodemographic variables	Baseline *n* = 194 *n* (%)	Implementation *n* = 366 *n* (%)	*p*-value	Sustainability *n* = 153 *n* (%)	*p*-value
Age group					
18–60 years	135 (69.59)	228 (62.30)	0.10	83 (54.25)	0.11
>60 years	59 (30.41)	138 (37.70)		70 (45.75)	
Biological sex					
Female	121 (62.37)	212 (57.92)	0.35	87 (56.86)	0.90
Male	73 (37.63)	154 (42.08)		66 (43.14)	
Social security system					
Contributory	177 (91.24)	312 (85.25)	0.09	125 (81.70)	0.60
State-subsidised	11 (5.67)	41 (11.20)		21 (13.73)	
Other	6 (3.09)	13 (3.55)		7 (4.58)	
Clinical variables					
Weight in kg					
Median (IQR)	67.50 (59.67–74.25)	67.00 (58.00–75.25)	0.93	66.00 (55.00–74.50)	0.23
Height in cm					
Median (IQR)	160.00 (154.00–168.00)	161.00 (155.00–168.00)	0.38	160.00 (155.00–167.00)	0.59
Length of hospital stay in days					
Median (IQR)	4.76 (2.25–10.81)	6.78 (4.04–13.78)	**<0.01**	7.96 (2.94–15.24)	0.98
Treating service					
General surgery	126 (64.95)	172 (46.99)	**<0.01**	66 (43.14)	0.13
Head and neck surgery	51 (26.29)	52 (14.21)		17 (11.11)	
Orthopedics and traumatology	1 (0.52)	37 (10.11)		24 (15.69)	
Gynecology and obstetrics	3 (1.55)	27 (7.38)		6 (3.92)	
Other	13 (6.70)	78 (21.31)		40 (26.14)	
Charlson index score					
Equal to 0	152 (78.35)	301 (82.24)	0.32	130 (84.97)	0.53
Greater than 0	42 (21.65)	65 (17.76)		23 (15.03)	
History of allergies					
Yes	33 (17.19)	48 (13.11)	0.24	22 (14.38)	0.81
No	159 (82.81)	318 (86.89)		131 (85.62)	
Number of medications administered					
1 medication	81 (41.75)	93 (25.41)	**<0.01**	33 (21.57)	0.43
2 medications	82 (42.27)	143 (39.07)		57 (37.25)	
3 or more medications	31 (15.98)	130 (35.52)		63 (41.18)	

Bold values: *p*-value indicating a statistically significant difference.

### 3.4 Clinical outcomes

No statistically significant differences were observed in the cumulative incidence of medication-related AEs across phases (baseline 4.12%; implementation 6.01%; sustainability 3.27%). Similarly, medication-related incidents remained stable (47.42%, 46.72%, and 50.98%, respectively). Most AEs were classified as potentially preventable, serious and associated with antibiotic use and prescribing errors. Most CIs were related to opioid use, particularly at the prescribing stage. Importantly, the absence of effect remained consistent during the sustainability phase (Table 4). The most common AE was impaired renal function. The most common CIs were administration of a non-prescribed medication, prescription of a non-indicated medication and missed therapeutic opportunities (Supplementary Table S2).

**Table 4:** Characterization of clinical outcomes, for each phase.

Characterization of AEs	Baseline *n* = 194 *n* (%)	Implementation *n* = 366 *n* (%)	*p*-value	Sustainability *n* = 153 *n* (%)	*p*-value
Cumulative incidence of AEs	8 (4.12)	22 (6.01)	0.45	5 (3.27)	0.29
Number of AEs reported	*n* = 8	*n* = 22		*n* = 5	
1	8 (100)	21 (95.45)	> 0.99	5 (100)	>0.99
2	0 (0.00)	1 (4.55)		0 (0.00)	
Median incidence proportion for AEs					
Median (IQR)	5.56 (2.70–5.77)	4.35 (2.82–8.96)	0.50	2.38 (0.00–5.41)	0.16
AEs preventability	*n* = 8	*n* = 23		*n* = 5	
Not potentially preventable	4 (50.00)	10 (43.48)	> 0.99	2 (40.00)	>0.99
Potentially preventable	4 (50.00)	13 (56.52)		3 (60.00)	
AEs classification					
Serious	8 (100)	18 (78.26)	0.38	4 (80.00)	>0.99
Not serious	0 (0.00)	5 (21.26)		1 (20.00)	
AEs associated medication					
Antibiotic	7 (87.50)	12 (52.17)	0.18	4 (80.00)	0.52
Opioid	1 (12.50)	11 (47.83)		1 (20.00)	
Cause of the error associated with the AE					
Administration error	1 (12.50)	0 (0.00)	0.10	0 (0.00)	0.96
Prescription error	5 (62.50)	20 (86.96)		5 (100)	
Adverse drug reaction	1 (12.50)	3 (13.04)		0 (0.00)	
Cause not recorded	1 (12.50)	0 (0.00)		0 (0.00)	
Characterization of CIs	Baseline *n* = 194 *n* (%)	Implementation *n* = 366 *n* (%)	*p*-value	Sustainability *n* = 153 *n* (%)	*p*-value
Cumulative incidence of CIs	92 (47.42)	171 (46.72)	0.95	78 (50.98)	0.43
Number of CIs reported	*n* = 92	*n* = 171		*n* = 78	
1	78 (84.78)	153 (89.47)	0.27	60 (76.92)	**0.04**
2	11 (11.96)	16 (9.36)		14 (17.95)	
3	3 (3.26)	1 (0.58)		3 (3.85)	
4	0 (0.00)	1 (0.58)		1 (1.28)	
Median incidence proportion for CIs					
Median (IQR)	47.37 (41.67–51.61)	47.06 (42.17–55.37)	0.91	50.00 (46.15–61.90)	0.66
Related stage with the CIs	*n* = 109	*n* = 192		*n* = 101	
Prescription	77 (70.64)	168 (87.50)	**0.01**	88 (87.13)	>0.99
Administration	32 (29.36)	24 (12.50)		13 (12.87)	
CIs associated medication					
Antibiotic	29 (26.61)	26 (13.54)	**<0.01**	25 (24.75)	**0.01**
Anticoagulant	15 (13.76)	15 (7.81)		14 (13.86)	
Opioid	65 (59.63)	151 (78.65)		62 (61.39)	

Bold values: *p*-value indicating a statistically significant difference.

**Figure 3:**
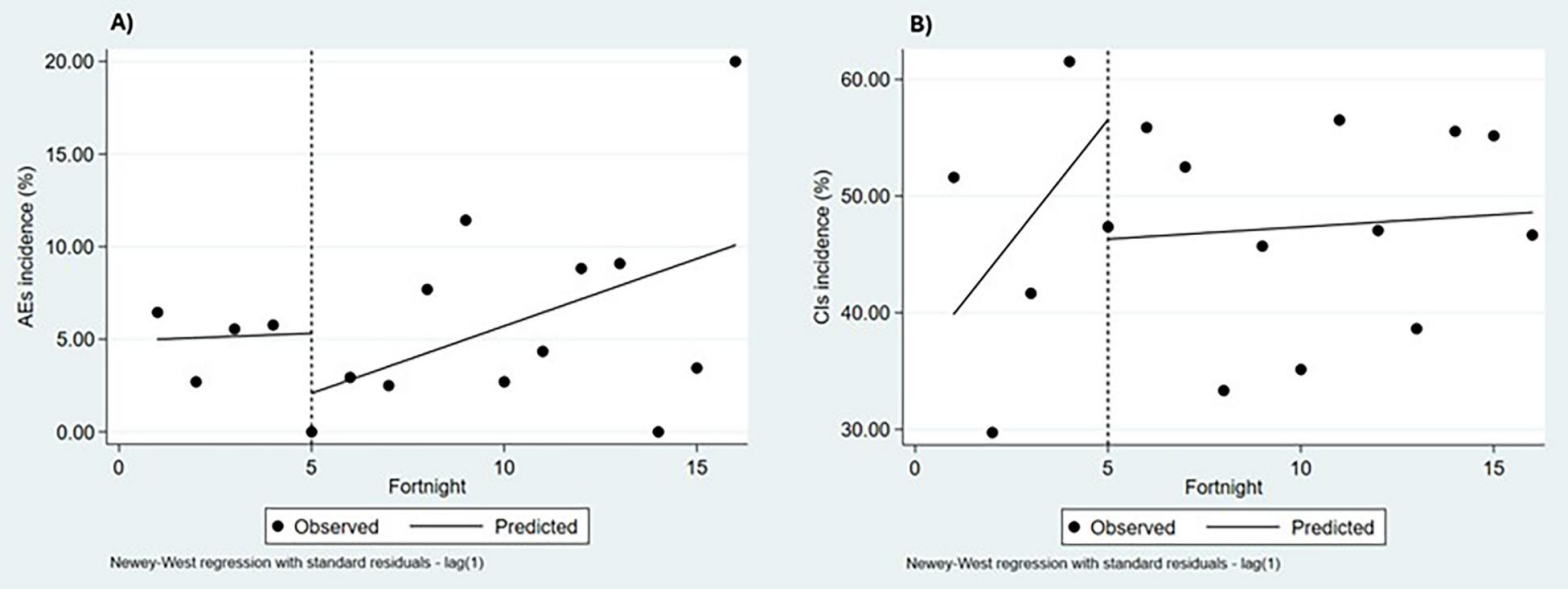
Interrupted time series of (**A**) AEs and (**B**) CIs under the newey-west regression model.

Segmented regression models using Newey–West standard errors showed no significant level or slope changes for AEs or CIs after intervention initiation (Table 5 and Figure 3). This result was corroborated after verifying the assumptions (Supplementary Tables S3–S5 and Supplementary Figures S8–S10) and adjusting the Durbin-Watson models for AEs and CIs (Supplementary Table S6).

**Table 5:** Results of the newey-west model adjusted for the AEs and CIs incidence.

Covariates	Newey-West model for the AEs incidence			Newey-West model for the CIs incidence		
	Coefficient	95% CI	*p*-value	Coefficient	95% CI	*p*-value
Intercept	4.92	(1.48; 8.36)	**0.01**	39.88	(20.08; 59.68)	**<0.01**
Trend in the incidence of the outcome over time before the start of the project (%)	0.08	(−1.00; 1.16)	0.87	4.17	(−5.45; 13.79)	0.36
Effect of the intervention (start of implementation of improvement ideas) on the incidence of the outcome (%)	−3.24	(−8.76; 2.28)	0.23	−10.25	(−36.95; 16.45)	0.42
Time-adjusted effect of intervention (start of implementation of improvement ideas) on the incidence of the outcome (%)	0.65	(−0.90; 2.19)	0.38	−3.97	(−13.72; 5.79)	0.39
Trend in outcome incidence over time after start of implementation (%)	0.73	(−0.42; 1.87)	0.19	0.21	(−1.17; 1.58)	0.75

Bold values: *p*-value indicating a statistically significant difference.

Adjusted logistic regression models confirmed absence of intervention effect: AEs: aOR=1.14 (95% CI; 0.46–2.83) and CIs: aOR=1.12 (95% CI: 0.76–1.65). Risk factors independently associated with outcomes included age >60 years for AEs and receiving 3 or more medications, general surgery service, and head and neck surgery for CIs (Supplementary Table S7).

### 3.5 Contextual influences on implementation

Implementation occurred within a complex academic hospital environment characterized by organizational fragmentation, workforce instability, and structural resource constraints. These contextual elements interacted dynamically with intervention delivery and likely constrained activation of intended mechanisms of change.

First, high turnover among surgical residents, anesthesiology trainees, and hospital-employed physicians created discontinuity in clinical practices. Rotational training models meant that newly arriving clinicians had limited exposure to prior improvement cycles and insufficient time to internalize protocol modifications or adopt new tools such as the PROA UNAL application. This reduced cumulative behavioral reinforcement and impeded sustained fidelity.

Second, governance fragmentation weakened accountability structures. Residents and some faculty members operated under university governance, whereas nursing and hospital physicians were administratively accountable to hospital leadership. This dual structure diluted responsibility for implementation oversight and reduced coordinated leadership reinforcement of new prescribing and monitoring practices.

Third, human resource limitations, particularly in postoperative recovery units, constrained operational feasibility. Nursing shortages limited consistent pain assessment documentation and real-time monitoring of medication administration practices. Because pain scale documentation was central to the opioid stewardship component, insufficient staffing directly reduced fidelity of that intervention mechanism.

Fourth, prescribing authority and clinical decision-making were distributed across professionals with variable communication patterns. Hospital physicians frequently executed medication orders defined by specialists or residents with limited direct coordination. This separation between prescribing intent and prescribing execution may have attenuated the intended effect of decision-support tools.

Fifth, limited integration of performance data into routine decision-making reduced adaptive capacity. Although process indicators were measured biweekly, data were not embedded into structured, real-time feedback systems. Consequently, opportunities for rapid course correction during PDSA cycles were constrained. In complex adaptive systems, timely feedback is often necessary to reinforce behavioral change; its absence may weaken intervention momentum.

Finally, cultural and hierarchical norms influenced engagement. Improvement initiatives relied on voluntary adoption of new practices rather than mandatory workflow integration. In settings where clinical autonomy is highly valued and improvement initiatives compete with heavy clinical workloads, voluntary behavioral modification may be insufficient to sustain change.

Collectively, these contextual factors interacted with implementation fidelity. Rather than representing isolated barriers, they formed a reinforcing system that limited exposure, reduced adherence, weakened accountability, and constrained adaptation. Under such conditions, the intervention was delivered inconsistently across providers and time periods, reducing the likelihood that intended causal mechanisms would be activated and translated into measurable clinical outcomes.

## 4 Discussion

### 4.1 Principal findings

This study evaluated a set of interventions under the CQI methodology aimed at improving prescribing and medication administration in surgical services and conducted a process assessment informed by MRC guidance to explain the absence of expected clinical effects.

Despite robust statistical design, including ITS analysis and multivariable adjustment, no significant changes were observed in process indicators, medication-related AEs, or medication-related CIs.

The process assessment suggests that limited implementation fidelity and contextual misalignment, rather than intrinsic ineffectiveness of the CQI methodology, explain these findings.

### 4.2 Mechanisms not activated

According to the intervention theory, improvement in medication safety required modification of prescribing and administration behaviors. However, no sustained changes were observed in process indicators.

In complex interventions, absence of change in proximal process measures typically precedes absence of effect in distal outcomes. Without consistent modification of prescribing timing, documentation practices, and decision-support utilization, downstream reductions in AEs and CIs were unlikely.

Thus, the intervention's core mechanisms were only partially activated.

### 4.3 Contextual constraints in LMICs surgical settings

Consistent with broader literature on quality improvement in LMICs (12), structural and organizational constraints played a critical role.

High workforce turnover limited continuity. Fragmented governance structures diluted accountability. Limited nursing capacity constrained monitoring. Additionally, absence of real-time data feedback limited adaptive course correction during implementation.

Similar null or modest effects have been described in other CQI initiatives (20–22), particularly when methodological rigor was not accompanied by sustained behavioral and organizational change.

In contrast, studies reporting successful reductions in medication-related harm (23) often describe prolonged implementation periods, stronger clinician engagement, and embedded cultural change.

Our findings highlight that the interventions implemented under the CQI as a methodology is contingent upon system readiness and alignment.

### 4.4 Methodological implications

This study demonstrates that effective interventions and methods of risk management are necessary but insufficient to ensure measurable impact if implementation fidelity is limited. Process-informed evaluation is essential to interpret null findings appropriately. Without examining fidelity, mechanisms, and context, null results may be misinterpreted as failure of the improvement method itself.

### 4.5 Lessons for future CQI initiatives

The findings of this study suggest that the effectiveness of the interventions implemented under the CQI methodology in complex surgical environments depends less on the theoretical soundness of the intervention and more on the alignment between intervention design, organizational readiness, and contextual stability. For future CQI efforts in LMICs hospital settings, several strategic considerations emerge.

First, formal assessment of organizational readiness should precede implementation. CQI interventions frequently assume that clinical teams possess sufficient workforce stability, governance alignment, and operational flexibility to absorb change. However, in settings characterized by high staff turnover, dual governance structures, and workforce shortages, these assumptions may not hold. Structured readiness assessments—evaluating leadership commitment, staffing continuity, data infrastructure, and workflow capacity—can help determine whether the system is prepared to support sustained behavioral change. Without such assessment, interventions risk being introduced into environments structurally incapable of sustaining them.

Second, governance alignment is critical for accountability and reinforcement. In this study, fragmented authority between university-affiliated clinicians and hospital-employed staff diluted ownership of the intervention. Effective CQI requires clearly defined leadership structures, explicit role accountability, and shared performance metrics across professional groups. Interventions should be embedded within formal institutional mandates rather than relying solely on voluntary adoption. When accountability mechanisms are unified, reinforcement of practice change becomes consistent and sustainable.

Third, CQI initiatives should incorporate real-time, actionable feedback systems. Although process indicators were collected biweekly, they were not integrated into structured, adaptive learning loops. In complex healthcare systems, behavior change is reinforced through visible, timely performance data that clinicians perceive as relevant to their daily practice. Dashboards, brief feedback meetings, and rapid-cycle evaluation mechanisms allow teams to detect early signals of stagnation and adjust strategies accordingly. Data that are delayed or disconnected from operational decision-making rarely generate sustained engagement.

Fourth, interventions should prioritize proximal and behaviorally sensitive process indicators before expecting distal clinical outcomes to change. Medication-related adverse events are influenced by multiple upstream and downstream factors. If proximal prescribing and documentation behaviors do not shift measurably, expecting immediate reductions in AEs and CIs may be unrealistic. Future CQI initiatives should identify high-leverage, observable process changes that are feasible within the local workflow and capable of demonstrating early momentum. Early wins reinforce engagement and increase the likelihood of downstream clinical impact.

Fifth, frontline engagement must move beyond informational sessions toward embedded clinical leadership. Educational meetings and tool dissemination are insufficient when competing with heavy clinical workloads and entrenched professional norms. Identifying clinical champions within each service, integrating improvement tasks into routine workflow rather than adding parallel responsibilities, and aligning CQI objectives with professional incentives may strengthen behavioral adoption. Ownership at the point of care is often the determining factor between procedural compliance and sustained cultural change.

Finally, intervention scope must match contextual capacity. Multicomponent interventions, while theoretically comprehensive, may overwhelm teams operating in constrained environments. Phased implementation, prioritizing one high-impact component before layering additional elements, may increase fidelity and allow adaptation based on early feedback. In LMICs settings, strategic simplicity may be more effective than simultaneous complexity.

Collectively, these recommendations emphasize that CQI and interventions implemented under its methodology success are not solely a function of methodological rigor or statistical power. It depends on system readiness, governance coherence, workforce stability, feedback integration, and sustained behavioral reinforcement. In complex surgical services within resource-constrained contexts, improvement science must be applied not only to clinical processes but also to the organizational conditions that enable those processes to change.

By explicitly examining the interaction between context, implementation fidelity, and mechanisms of impact, this study contributes to a more nuanced understanding of why well-designed interventions or CQI projectss may fail to achieve measurable outcomes. Such understanding is essential to avoid prematurely discarding improvement methodologies that, when appropriately aligned with context, retain substantial potential to enhance patient safety.

### 4.6 Strengths and limitations

Strengths of our study include robust statistical modeling, random sampling, absence of loss to follow-up, and adjustment for confounding variables.

Limitations include partial implementation fidelity, limited number of time points, and contextual instability affecting sustainability. Performance bias due to incomplete adherence likely attenuated measurable effects toward the null.

## 5 Conclusions

This study found no measurable reduction in medication-related AEs or CIs following implementation of a set of interventions under the CQI methodology in surgical services.

A process evaluation informed by MRC guidance suggests that limited implementation fidelity, workforce instability, fragmented governance, and insufficient real-time data integration constrained activation of intended mechanisms of change.

These findings underscore that CQI interventions in LMICs surgical settings require structural readiness, sustained engagement, and contextual alignment to translate methodological rigor into meaningful patient safety improvements.

## Data Availability

The raw data supporting the conclusions of this article will be made available by the authors, without undue reservation.
