# Supplementary material for: Understanding limited effectiveness of a set of interventions implemented under continuous quality improvement methodology: a process assessment informed by MRC guidance
Source: Front Health Serv. 2026 Jul 15;6:1884875. doi: 10.3389/frhs.2026.1884875 (PMC13416563; doi:10.3389/frhs.2026.1884875)
Supplement: Supplementary file 1 [file Datasheet1.docx]

Supplementary Material

# 2 Materials and Methods

## 2.3 Intervention theory and components

**
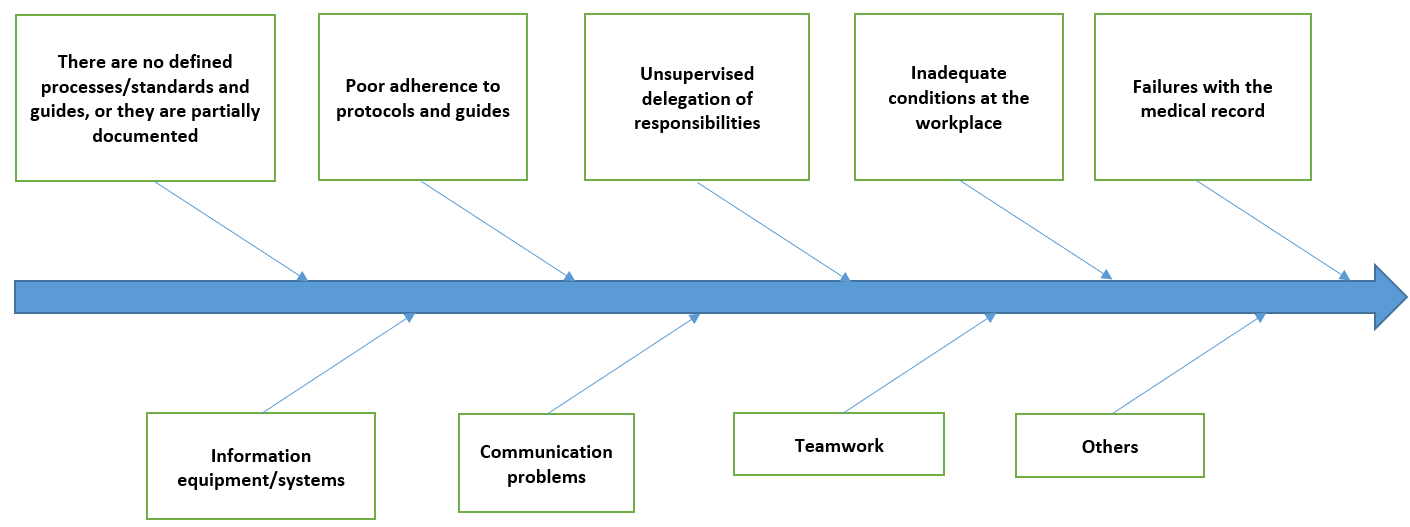
**

Supplementary Figure S1. Fishbone Diagram used in the root cause analysis

## 2.6 Outcomes

Supplementary Table S1. Definitions of drug process indicators

| **Medication type** | **Indicator number** | **Indicator name** | **Indicator formula** |
| --- | --- | --- | --- |
| Antibiotic | 1 | Proportion of patients with correct prescription of prophylactic antibiotics | Number of patients who had a correct prescription for prophylactic antibiotics (drug, dose, route, frequency) |
|  |  |  | Total number of patients with indication (institutional guideline or protocol) who underwent surgery |
|  | 2 | Proportion of patients who received opportunely the prophylactic antibiotic | Number of patients who received opportunely the prophylactic antibiotic and underwent surgery |
|  |  |  | Total number of patients who received prophylactic antibiotics and who underwent surgery |
| Anticoagulant | 3 | Proportion of patients with correct prescription of thromboprophylaxis | Number of patients with correct prescription of thromboprophylaxis (drug, dose, route, frequency, and duration) |
|  |  |  | Total number of patients with indication of thromboprophylaxis |
|  | 4 | Proportion of patients who received thromboprophylaxis | Number of patients who received thromboprophylaxis |
|  |  |  | Total number of patients with prescription of thromboprophylaxis |
| Opioid | 5 | Proportion of patients with a VAS pain score less than 4 | Number of patients with a VAS pain score of less than 4 |
|  |  |  | Total number of patients administered opioids, with indications for pain |
|  | 6 | Proportion of patients with a VAS pain score greater than or equal to 4 | Number of patients with a VAS pain score greater than or equal to 4 |
|  |  |  | Total number of patients administered opioids, with indications for pain |

# Results

## 3.2 Activation of mechanisms of impact

**
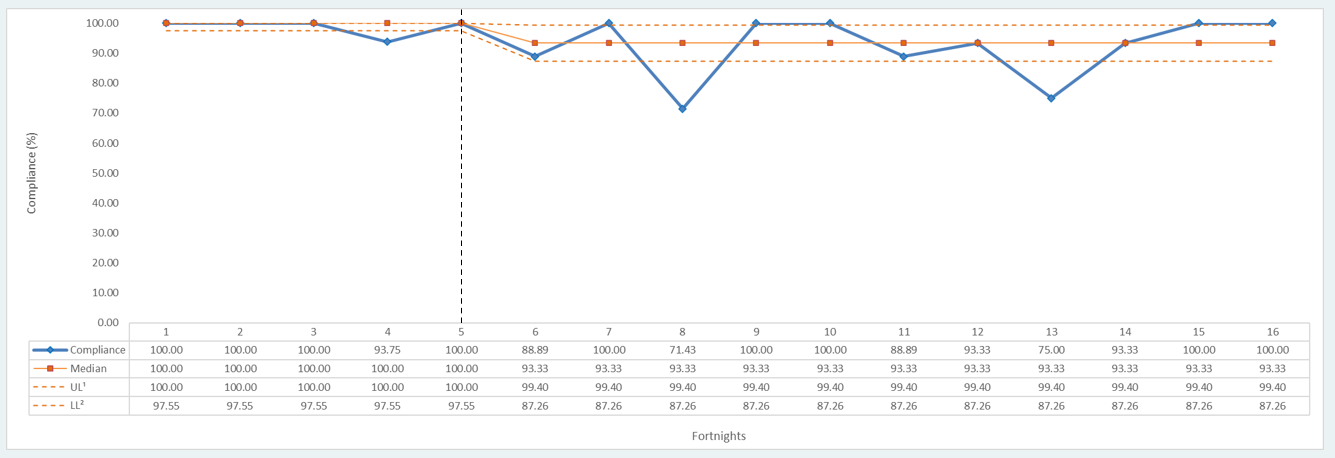
**

Supplementary Figure S2. Proportion of patients with correct prescription of prophylactic antibiotics

Abbreviations: ^1^UL: Upper Limit; ^2^LL: Lower Limit

**
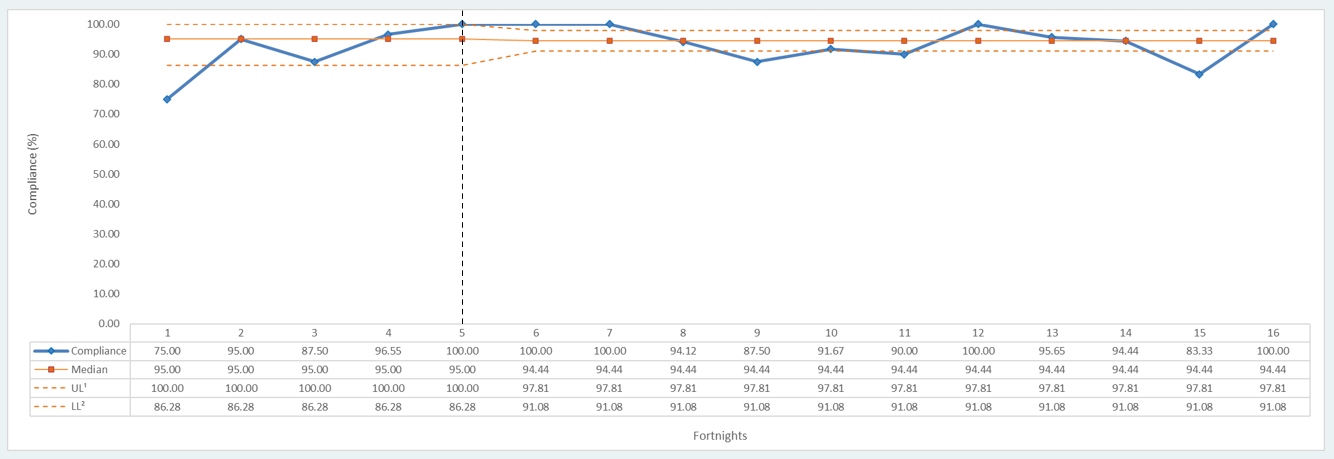
**

Supplementary Figure S3. Proportion of patients who received opportunely the prophylactic antibiotic

Abbreviations: ^1^UL: Upper Limit; ^2^LL: Lower Limit

**
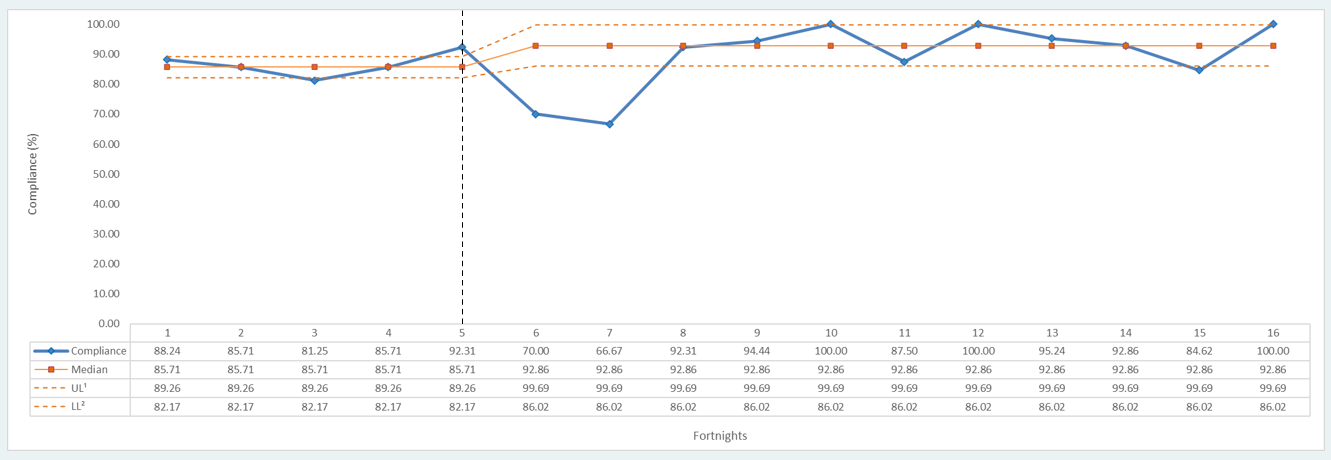
**

Supplementary Figure S4. Proportion of patients with correct prescription of thromboprophylaxis

Abbreviations: ^1^UL: Upper Limit; ^2^LL: Lower Limit

**
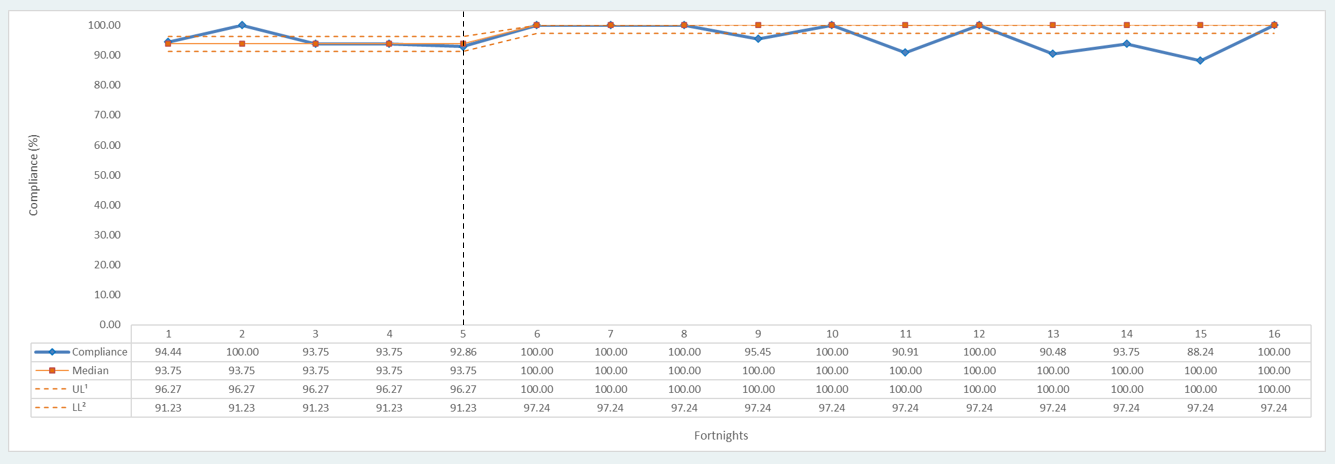
**

Supplementary Figure S5. Proportion of patients who received thromboprophylaxis

Abbreviations: ^1^UL: Upper Limit; ^2^LL: Lower Limit

**
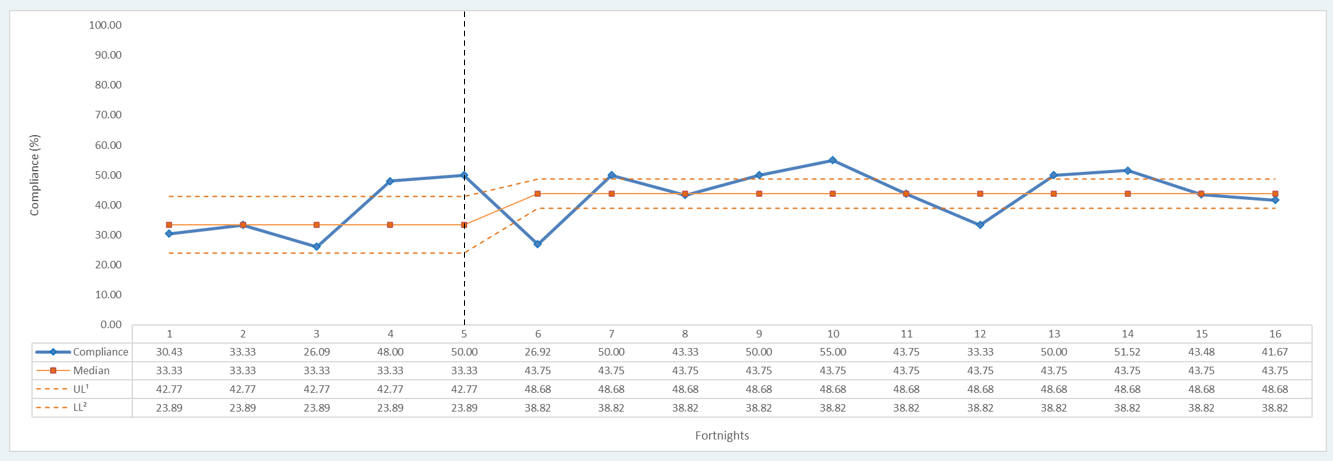
**

Supplementary Figure S6. Proportion of patients with a VAS pain score less than 4

Abbreviations: ^1^UL: Upper Limit; ^2^LL: Lower Limit

**
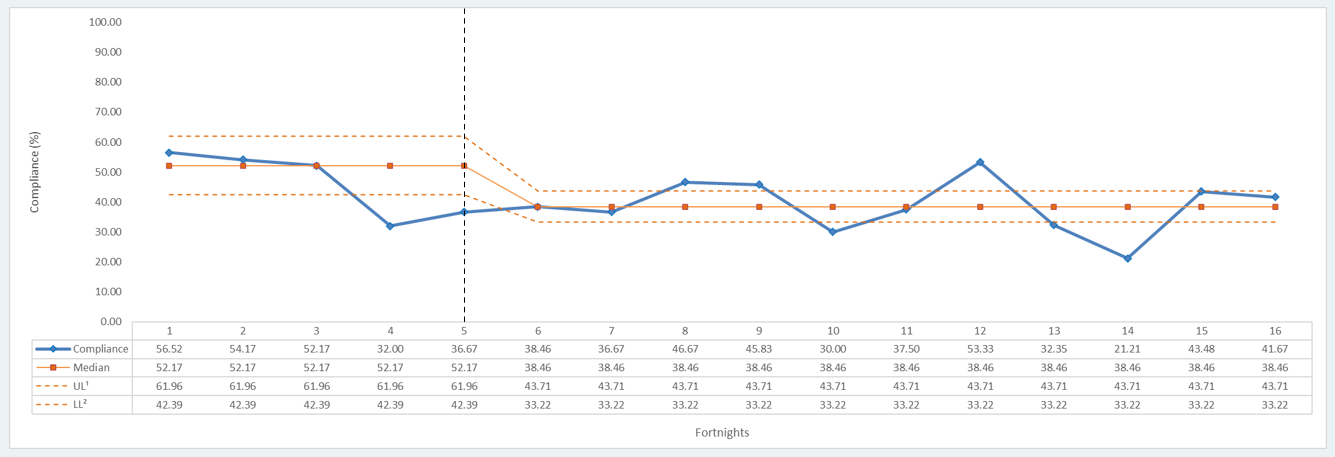
**

Supplementary Figure S7. Proportion of patients with a VAS pain score greater than or equal to 4

Abbreviations: ^1^UL: Upper Limit; ^2^LL: Lower Limit

## Clinical outcomes

Supplementary Table S2. Type and frequency of AEs and CIs by phase

|  | **Baseline**  **n = 8**  **n (%)** | **Implementation**  **n = 23**  **n (%)** | **p-value** | **Sustainability**  **n = 5**  **n (%)** | **p-value** |
| --- | --- | --- | --- | --- | --- |
| **AEs name** |  |  |  |  |  |
| Bradycardia requiring medication | 0 (0.00) | 2 (8.70) | 0.51 | 0 (0.00) | **0.02** |
| Drop in blood pressure + Hypotension | 1 (12.50) | 4 (17.39) |  | 0 (0.00) |  |
| Convulsion | 0 (0.00) | 1 (4.35) |  | 0 (0.00) |  |
| Desaturation (possible respiratory depression) | 0 (0.00) | 0 (0.00) |  | 1 (20.00) |  |
| Diarrhea | 0 (0.00) | 1 (4.35) |  | 0 (0.00) |  |
| Impaired renal function | 5 (62.50) | 8 (34.78) |  | 0 (0.00) |  |
| Medullary brake | 0 (0.00) | 1 (4.35) |  | 0 (0.00) |  |
| Kidney injury | 2 (25.00) | 1 (4.35) |  | 3 (60.00) |  |
| Allergic reaction | 0 (0.00) | 1 (4.35) |  | 1 (20.00) |  |
| Sleepiness | 0 (0.00) | 4 (17.39) |  | 0 (0.00) |  |
| **CIs name** | **n = 109** | **n = 192** |  | **n = 101** |  |
| Administration of non-prescribed medication | 62 (56.88) | 152 (79.17) | > 0.99 | 65 (64.36) | > 0.99 |
| Changes in vital signs associated with medication (hypotension, bradycardia, dyspnea, among others) | 3 (2.75) | 4 (2.08) |  | 1 (0.99) |  |
| Dismissed | 0 (0.00) | 1 (0.52) |  | 1 (0.99) |  |
| Formulation of medication with contraindication | 0 (0.00) | 1 (0.52) |  | 0 (0.00) |  |
| Formulation of medication after administration | 2 (1.83) | 0 (0.00) |  | 2 (1.98) |  |
| Inconsistency between the plan and the formula | 2 (1.83) | 5 (2.60) |  | 1 (0.99) |  |
| Inconsistency between the prescribed and administered doses | 1 (0.92) | 2 (1.04) |  | 4 (3.96) |  |
| There is no record of anesthesia | 1 (0.92) | 0 (0.00) |  | 0 (0.00) |  |
| There is no administration record | 10 (9.17) | 7 (3.65) |  | 0 (0.00) |  |
| No medication formula is identified | 0 (0.00) | 2 (1.04) |  | 0 (0.00) |  |
| No medication is prescribed | 0 (0.00) | 0 (0.00) |  | 1 (0.99) |  |
| Prescribed opioid not in accordance with VAS | 0 (0.00) | 1 (0.52) |  | 1 (0.99) |  |
| Missed therapeutic opportunities | 17 (15.60) | 4 (2.08) |  | 3 (2.97) |  |
| Prescription of medication with incorrect dosage | 2 (1.83) | 4 (2.08) |  | 2 (1.98) |  |
| Prescription of a non-indicated medication | 6 (5.50) | 5 (2.60) |  | 15 (14.85) |  |
| Skin reaction associated with administered medication | 0 (0.00) | 0 (0.00) |  | 1 (0.99) |  |
| Administration record frequently incorrect | 0 (0.00) | 1 (0.52) |  | 0 (0.00) |  |
| Administration record of suspended medication | 1 (0.92) | 0 (0.00) |  | 0 (0.00) |  |
| Administration record of wrong medication | 1 (0.92) | 0 (0.00) |  | 0 (0.00) |  |
| Incomplete administration record | 0 (0.00) | 1 (0.52) |  | 0 (0.00) |  |
| Incorrect administration record | 1 (0.92) | 0 (0.00) |  | 0 (0.00) |  |
| Gastric symptoms (nausea, vomiting, diarrhea, constipation) | 0 (0.00) | 1 (0.52) |  | 1 (0.99) |  |
| Neurological symptoms (phosphenes, tinnitus, vertigo, drowsiness) | 0 (0.00) | 1 (0.52) |  | 3 (2.97) |  |

Supplementary Table S3. Autocorrelation and partial autocorrelation for the AEs and CIs incidence

|  | **AEs incidence** | | | **CIs incidence** | | |
| --- | --- | --- | --- | --- | --- | --- |
| **Lag** | Autocorrelation | Partial autocorrelation | p-value | Autocorrelation | Partial autocorrelation | p-value |
| 1 | -0.04 | -0.11 | 0.85 | -0.13 | -0.13 | 0.57 |
| 2 | -0.37 | -0.88 | 0.23 | -0.21 | -0.23 | 0.53 |
| 3 | 0.15 | 0.50 | 0.33 | 0.05 | -0.02 | 0.73 |
| 4 | 0.20 | 0.18 | 0.36 | -0.34 | -0.45 | 0.39 |
| 5 | -0.05 | 0.03 | 0.48 | 0.06 | -0.10 | 0.51 |
| 6 | -0.24 | -0.62 | 0.41 | -0.05 | -0.51 | 0.63 |

**
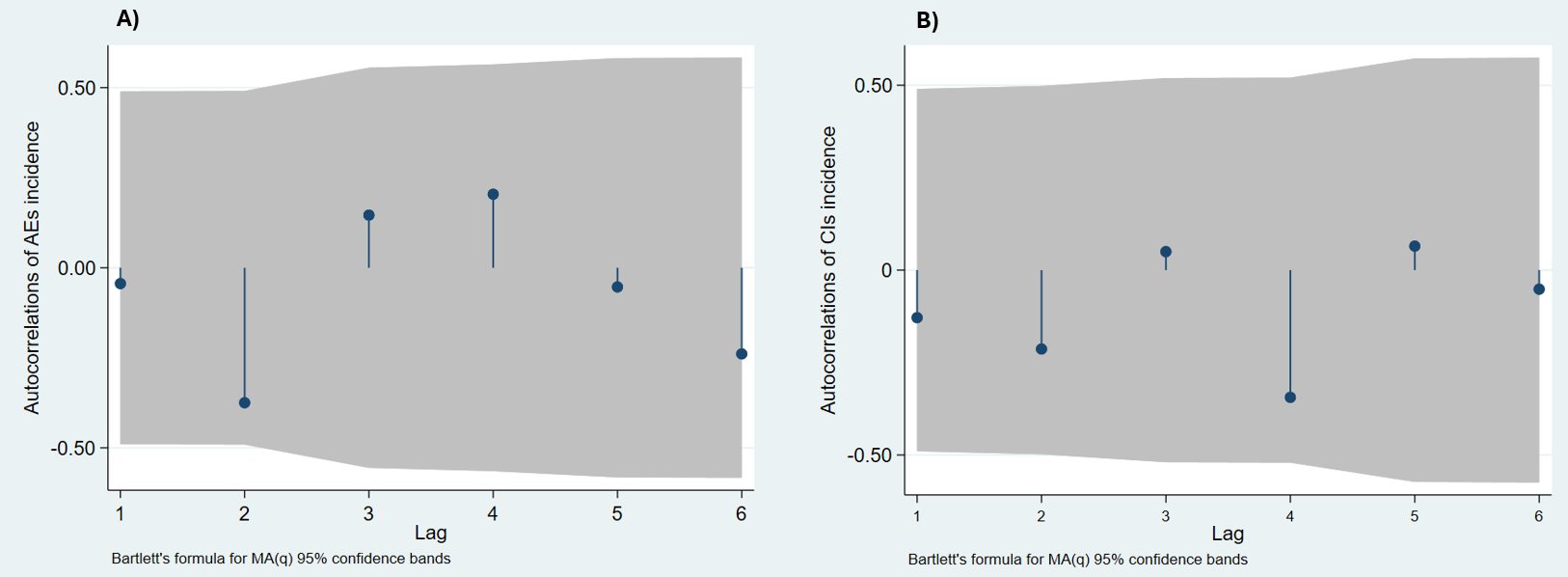
**

Supplementary Figure S8. Autocorrelogram for the A) AEs and B) CIs incidence

Abbreviations: MA(q): q-order moving average

Supplementary Table S4. Autocorrelation and partial autocorrelation for the residuals of the Newey-West model for AEs and CIs

|  | **AEs incidence** | | | **CIs incidence** | | |
| --- | --- | --- | --- | --- | --- | --- |
| **Lag** | Autocorrelation | Partial autocorrelation | p-value | Autocorrelation | Partial autocorrelation | p-value |
| 1 | -0.07 | -0.11 | 0.77 | -0.20 | -0.20 | 0.37} |
| 2 | -0.49 | -0.93 | 0.08 | -0.22 | -0.25 | 0.41} |
| 3 | 0.08 | 0.29 | 0.16 | 0.05 | -0.04 | 0.61 |
| 4 | 0.19 | 0.03 | 0.20 | -0.29 | -0.43 | 0.42 |
| 5 | -0.15 | -0.36 | 0.25 | 0.15 | -0.03 | 0.47 |
| 6 | -0.35 | -1.26 | 0.12 | 0.02 | -0.23 | 0.60 |

**
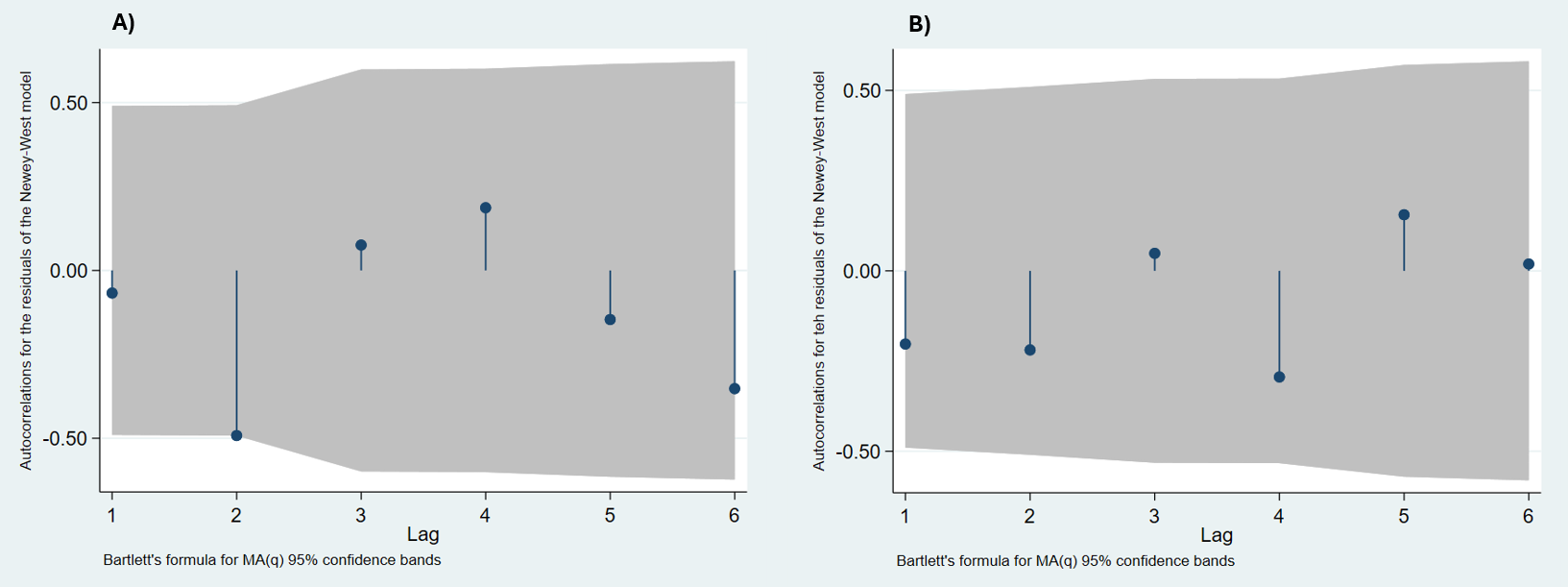
**

Supplementary Figure S9. Autocorrelogram for the residuals of the Newey-West model for A) AEs and B) CIs

Abbreviations: MA(q): q-order moving average

**
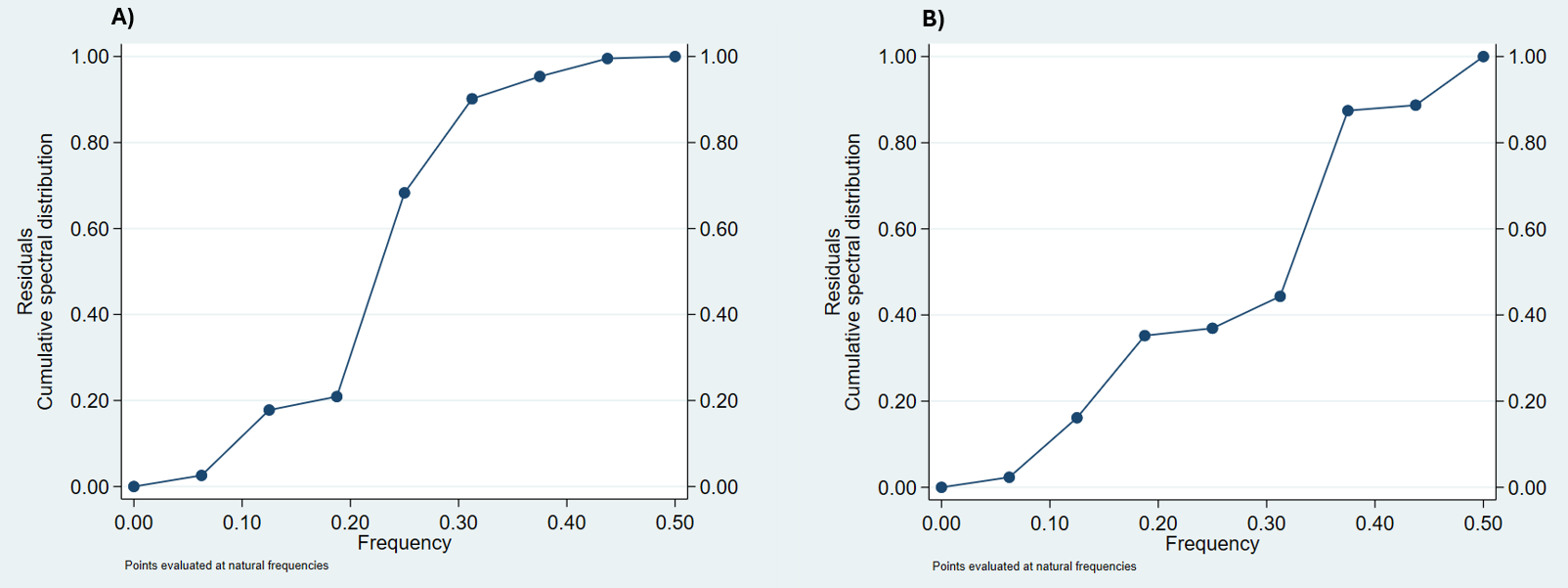
**

Supplementary Figure S10. Cumulative periodogram for the residuals of the Newey-West model for A) AEs and B) CIs

Supplementary Table S5. Results of the Bartlett and Cumby-Huizinga test for the residuals corresponding to the Newey-West regression model adjusted for AEs and CIs

|  | **AEs incidence** | | | | **CIs incidence** | | | |
| --- | --- | --- | --- | --- | --- | --- | --- | --- |
| **Bartlett test** | $\mathsf{\chi}^{2}$ | | p-value | | $\mathsf{\chi}^{2}$ | | p-value | |
|  | 3.36 | | 0.07 | | 0.89 | | 0.34 | |
| **Cumby-Huizinga test** | Autocorrelation over a range of lags | | Autocorrelation in a specific lag | | Autocorrelation over a range of lags | | Autocorrelation in a specific lag | |
|  | Lag | p-value | Lag | p-value | Lag | p-value | Lag | p-value |
|  | 1-1 | 0.71 | 1 | 0.71 | 1-1 | 0.39 | 1 | 0.39 |
|  | 1-2 | **0.02** | 2 | **0.01** | 1-2 | 0.32 | 2 | 0.38 |
|  | 1-3 | **0.04** | 3 | 0.63 | 1-3 | 0.51 | 3 | 0.83 |
|  | 1-4 | 0.09 | 4 | 0.24 | 1-4 | 0.29 | 4 | 0.22 |
|  | 1-5 | 0.14 | 5 | 0.33 | 1-5 | 0.40 | 5 | 0.56 |
|  | 1-6 | **0.04** | 6 | **0.01** | 1-6 | 0.36 | 6 | 0.94 |

Supplementary Table S6. Results of the Durbin-Watson model adjusted for the AEs and CIs incidence

|  | **Durbin-Watson model for the AEs incidence** | | | **Durbin-Watson model for the CIs incidence** | | |
| --- | --- | --- | --- | --- | --- | --- |
| **Covariates** | **Coefficient** | **95% CI** | **p-value** | **Coefficient** | **95% CI** | **p-value** |
| Intercept | 4.87 | (0.17; 9.58) | **0.04** | 38.28 | (15.72; 60.84) | **<0.01** |
| Trend in the incidence of the outcome over time before the start of the project (%) | 0.07 | (-1.30; 1.44) | 0,92 | 4.63 | (-6.46; 15.71) | 0.38 |
| Effect of the intervention (start of implementation of improvement ideas) on the incidence of the outcome (%) | -2.93 | (-8.51; 2.64) | 0,27 | -10.14 | (-39.08; 18.79) | 0.46 |
| Time-adjusted effect of intervention (start of implementation of improvement ideas) on the incidence of the outcome (%) | 0.62 | (-1.13; 2.37) | 0,46 | -4.45 | (-15.49; 60.84) | 0.40 |
| ρ | -0.08 | | | -0.22 | | |
| Original Durbin-Watson statistic | 1.79 | | | 2.29 | | |
| Transformed Durbin-Watson statistic | 1.80 | | | 1.96 | | |

Supplementary Table S7. Results of the logistic regression models adjusted for AEs and CIs

|  | **Logistic regression model for AEs** | | | | | | **Logistic regression model for CIs** | | | | | |
| --- | --- | --- | --- | --- | --- | --- | --- | --- | --- | --- | --- | --- |
| **Covariates** | **OR** | **95% CI** | **p-value** | **aOR** | **95% CI** | **p-value** | **OR** | **95% CI** | **p-value** | **aOR** | **95% CI** | **p-value** |
| Intercept | 0.06 | (0.04-0.08) | **< 0.01** | 0.01 | (0.00-0.11) | **< 0.01** | 0.89 | (0.75-1.05) | 0.15 | 0.22 | (0.07-0.72) | **0.01** |
| Implementation phase | 1.49 | (0.65-3.40) | 0.35 | 1.14 | (0.46-2.83) | 0.79 | 0.97 | (0.69-1.38) | 0.87 | 1.12 | (0.76-1.65) | 0.56 |
| Male | 1.99 | (0.95-4.19) | 0.07 | 1.62 | (0.73-3.56) | 0.23 | 0.87 | (0.62-1.22) | 0.43 | 0.99 | (0.68-1.43) | 0.95 |
| >60 years age group | 2.54 | (1.21-5.35) | **0.01** | 2.22 | (1.00-4.92) | **0.05** | 0.87 | (0.61-1.23) | 0.42 | 0.80 | (0.55-1.17) | 0.25 |
| Length of hospital stay in days | 1.02 | (1.00-1.03) | **0.01** | 1.01 | (1.00-1.03) | 0.13 | 0.99 | (0.98-1.00) | 0.30 | 0.99 | (0.98-1.01) | 0.33 |
| Charlson index score | 1.06 | (0.72-1.57) | 0.77 | 0.95 | (0.63-1.44) | 0.82 | 1.09 | (0.90-1.32) | 0.36 | 1.13 | (0.93-1.39) | 0.23 |
| Number of medications administered |  |  |  |  |  |  |  |  |  |  |  |  |
| 2 medications | 2.18 | (0.68-6.98) | 0.19 | 2.01 | (0.62-6.53) | 0.25 | 1.15 | (0.78-1.72) | 0.48 | 1.37 | (0.90-2.08) | 0.14 |
| 3 or more medications | 4.37 | (1.42-13.45) | **0.01** | 3.03 | (0.91-10.13) | 0.07 | 1.40 | (0.91-2.16) | 0.12 | 2.08 | (1.25-3.46) | **0.01** |
| Social security system |  |  |  |  |  |  |  |  |  |  |  |  |
| Contributory | 0.93 | (0.12-7.25) | 0.94 | 1.89 | (0.19-18.56) | 0.59 | 1.51 | (0.58-3.90) | 0.40 | 1.16 | (0.41-3.27) | 0.77 |
| State-subsidised | 1.91 | (0.21-17.54) | 0.57 | 2.83 | (0.26-31.36) | 0.40 | 1.85 | (0.63-5.45) | 0.26 | 1.83 | (0.57-5.81) | 0.31 |
| Treating service |  |  |  |  |  |  |  |  |  |  |  |  |
| General surgery | 0.77 | (0.31-1.91) | 0.58 | 1.01 | (0.38-2.72) | 0.98 | 2.40 | (1.44-3.99) | **0.01** | 2.69 | (1.57-4.60) | **< 0.01** |
| Head and neck surgery | 0.24 | (0.05-1.17) | 0.08 | 0.51 | (0.09-2.81) | 0.44 | 3.49 | (1.91-6.36) | **< 0.01** | 4.56 | (2.36-8.81) | **< 0.01** |
| Orthopedics and traumatology | 0.67 | (0.13-3.37) | 0.62 | 0.59 | (0.11-3.17) | 0.54 | 1.63 | (0.74-3.61) | 0.23 | 1.47 | (0.65-3.33) | 0.35 |
| Gynecology and obstetrics | 0.41 | (0.05-3.51) | 0.42 | 0.54 | (0.06-5.17) | 0.60 | 2.86 | (1.22-6.68) | **0.02** | 2.40 | (0.97-5.97) | 0.06 |
